# Unlocking associative nitrogen fixation in Poaceae: molecular mechanisms, regulatory constraints, and pathways to sustainable productivity

**DOI:** 10.3389/fmicb.2026.1922705

**Published:** 2026-10-05

**Authors:** Qian Nong, Qiang Liang, Xing Huang, Li Lin, Anjali Chandrol Solanki, Mukesh Kumar Malviya, Manoj Kumar Solanki, Krishan K. Verma, Changning Li

**Affiliations:** 1 Key Laboratory of Sugarcane Biotechnology and Genetic Improvement (Guangxi), Ministry of Agriculture and Rural Affairs, Nanning, Guangxi, China; 2 Department of Agriculture, SAM Global University, Raisen, Madhya Pradesh, India; 3 Institute of Sciences, SAGE University, Indore, Madhya Pradesh, India; 4 Department of Life Sciences and Biological Sciences, IES University, Bhopal, Madhya Pradesh, India

**Keywords:** associative nitrogen fixation, microbial engineering, microbiome engineering, nitrogenase, sustainable agriculture, synthetic communities

## Abstract

Biological nitrogen fixation (BNF) is a sustainable and environmentally friendly approach to nitrogen assimilation that extends beyond leguminous plants, where efficient nitrogen fixation occurs. Poaceae crops can also obtain nitrogen through associative nitrogen fixation (ANF) mediated by endophytic and rhizospheric diazotrophs, which typically contributes 10–30 kg N ha^−1^ yr.^−1^ to cereal crops, i.e., wheat, maize, rice, though certain tropical grasses, such as sugarcane may achieve up to 40–50 kg N ha^−1^ yr.^−1^ under optimal conditions. This review synthesizes recent advancements of ANF research across Poaceae crops, providing comprehensive analysis of their functional mechanisms. It explores the underlying mechanisms regulating ANF and assesses key factors influencing nitrogenase activity, including oxygen sensitivity, external nitrogen availability, and energy constraints. Furthermore, it discusses advanced strategies, such as genetic engineering of nitrogen-fixing microbes, host plant metabolic modifications, and optimized agronomic strategies to enhance nitrogen fixation efficacy. This review establishes a robust framework for sustainable crop production and long-term agricultural productivity by integrating molecular, physiological, and ecological perspectives. The major challenges of climate change, regional fertilizer restrictions, and achieving sustainable agricultural intensification collectively underscore the urgent need for biologically based nitrogen management strategies in cereal cropping systems.

## 1 Introduction

Nitrogen (N) is an important element for plant growth and development, and a major limiting factor for plant production. Nitrogen is essential for numerous physiological processes, such as protein, nucleic acid, chlorophyll synthesis, plant hormone production, and energy metabolism. However, most plants cannot directly utilize atmospheric nitrogen (N₂), which constitutes 78% of the air and instead depend on soil-derived bioavailable N to meet their nutritional demands (Priyadarshini et al., 2021). Thus, biological nitrogen fixation (BNF), a microbial process in which nitrogen-fixing microorganisms enzymatically convert atmospheric N into ammonia, plays a crucial role in the N cycle. The legume–rhizobium symbiosis contributes nearly 55 Mts of fixed N annually, accounting for about 55% of total BNF in terrestrial ecosystems. Biological nitrogen fixation is the most efficient nitrogen-fixing system in nature and is fundamental to maintaining nitrogen balance in ecosystems (Magori and Kawaguchi, 2009). The BNF system is classified into three categories, such as the free-living nitrogen fixation (FNF), symbiotic nitrogen fixation (SNF), and associative nitrogen fixation (ANF) (Xu and Wang, 2023).

The FNF occurs in specific prokaryotic microorganisms, including cyanobacteria and *Nostoc*, which independently fix nitrogen. These microorganisms typically possess photosynthetic and nitrogen-fixing capabilities, generating energy through photosynthesis to meet the energetic demands of N fixation, thereby sustaining their growth (Pankievicz et al., 2019; Soumare et al., 2020). The SNF is a mutualistic relationship between rhizobia and leguminous plants, in which rhizobia specifically recognize legume hosts and induce root nodule formation. Within these nodules, rhizobia differentiate into Bacteroides, which perform nitrogen fixation. The host plant supplies carbon sources through photosynthesis to support Bacteroide growth, development, and N fixation, while Bacteroides provide fixed N for plant development (Zhong et al., 2023). In contrast, diazotrophic bacteria mediate ANF with a broad host range, including *Oryza sativa*, *Zea mays*, *Triticum aestivum*, and *Saccharum officinarum*. The ANF bacteria derive nutrients from the root exudates to sustain their growth and N fixation without forming specialized symbiotic structures with host plants (Pankievicz et al., 2019; Soumare et al., 2020). Unlike the highly coordinated nodule-based symbiosis in legumes, ANF involves plant–microbe interactions strongly influenced by the host genotype, soil micro-environment, and microbial community dynamics. Thus, the complexity of ANF makes it more challenging to optimize, yet it holds more promise for sustainable cereal crop production.

The growing global population, currently approximately 8.2 billion and projected to reach 10.3 billion by the mid-2080s, is intensifying demand for food and water while facing constraints from limited arable land. The key strategy to address this challenge is enhancing food production. Nonetheless, optimum use of inorganic fertilizers to increase yield can contaminate soil and cause pollution (Ahmed et al., 2017). Therefore, BNF presents a promising and sustainable N source. The N fixation efficiency of cereal crops are lower than legumes, but their extensive cultivation area suggests an underestimated overall N fixation potential (Li et al., 2023; Yan et al., 2022; Zhang et al., 2022; Aasfar et al., 2024). For instance, recent omics-based approaches (metagenomics, transcriptomics, and metabolomics) have revealed the hidden diversity of diazotrophs in grasses microbiomes, as well as novel pathways regulating nitrogenase activity and plant–microbe interactions (Solanki et al., 2020; Malviya et al., 2021; Jain et al., 2024; Xiong et al., 2024). These findings underscore the need for integrative approaches to understand ANF at multiple biological scales. Thus, synthetic microbial communities (SynComs) have emerged as powerful tools for dissecting plant–microbe interactions and designing inoculants with predictable outcomes. Unlike single-strain inoculants, SynComs enable the reconstruction of core diazotrophic taxa in controlled systems, allowing for reproducible insights into colonization dynamics, nitrogenase activity, and cross-kingdom signaling. Shayanthan et al. (2022) highlights the potential of SynComs to bolster crop production by stabilizing microbiome functionality through synergistic interactions between members, the functional expression of nitrogenase components in transgenic rice (Baysal et al., 2022; He et al., 2022; Guo et al., 2023a).

Host-microbe syntrophy, represents the reciprocal and co-regulated metabolic interdependence between the grasses host plants and its associated diazotrophic microbiome, wherein plant-derived carbon substrates, i.e., root exudates, mucilage drive microbial nitrogen fixation, and microbially fixed nitrogen alongside phytohormones and other growth factors stimulates further host photosynthate production and exudate release (Korenblum et al., 2022; Halshoy et al., 2025). This bidirectional carbon–nitrogen (C-N) metabolic coupling is reinforced by host-conditioned physicochemical niches and microbe-responsive plant gene expression, rendering the partnership more than simple mutualism and approaching a state of obligate metabolic co-dependence under nitrogen-limited conditions (Xie et al., 2020).

Furthermore, major global initiatives, such as the C_4_ Rice Project and the Bill and Melinda Gates Foundation, invest in-depth research to enhance N fixation in staple crops.^1^ These efforts aim to reduce dependency on synthetic fertilizers, improve yield stability under low-N inputs, and contribute to climate-smart agriculture. Understanding the mechanisms that regulate ANF in cereal crops are crucial for increasing N fixation and reducing fertilizer dependence, which is key to promoting the sustainable development of ecosystems and mitigating environmental pollution from excessive fertilizer use (Figure 1). This review examines associative nitrogen-fixing bacteria in wheat, rice, maize, and sugarcane, focusing on their diversity, functional mechanisms, regulatory pathways, and potential agricultural applications.

**Figure 1:**
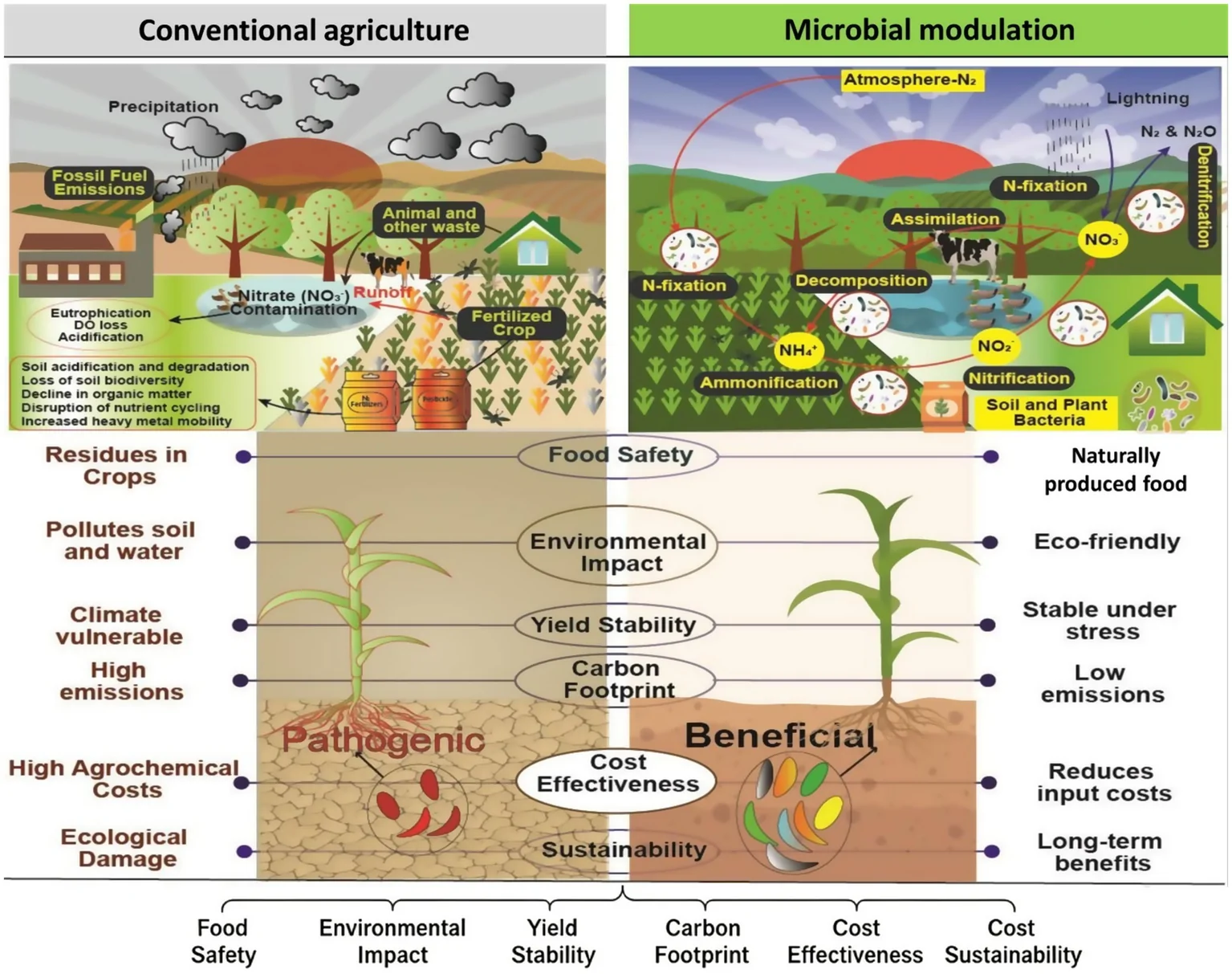
Comparison of conventional and microbe-mediated farming in relation to N cycling and plant-microbe interactions. Conventional agriculture leads to nitrate (NO₃^−^) contamination, soil acidification, and emissions, whereas microbe-mediated farming promotes N-fixation, enhanced nutrient uptake and sustainability. N = nitrogen; NO₃− = nitrate.

## 2 Major groups of nitrogen-fixing bacteria and significant impact

Nitrogen-fixing bacteria of cereal crops were first discovered in 1961 when Dobereiner isolated the N-fixing bacterium *Beijerinckia* from sugarcane (Dobereiner, 1961). The symbiotic relationship between cereal crops and N-fixing bacteria supplies N directly for plant uptake beside regulating and enhancing N conversion and metabolism of exogenous through various metabolic pathways, thereby improving N absorption and utilization efficiency (NUE) (Yan et al., 2022; Liu et al., 2024). The physiological and ecological characteristics categorize the N-fixing bacteria of cereal crops into rhizosphere and endophytic N-fixing microorganisms, each colonizing distinct ecological niches and contributing to N supply. Diazotrophic communities in grasses are primarily shaped by environmental factors rather than host, which is mainly comprised the taxa *Azospirillum* and *Methylobacterium* (Xiong et al., 2024). Metagenomic analyses revealed novel MAGs (metagenome-assembled genomes) with diverse N metabolism, including complete denitrification genes, expanding the understanding of phyllosphere N cycling (Xiong et al., 2024). Rhizosphere N-fixing bacteria, such as *Azotobacter paspali* and *Beijerinckia* spp., primarily enhance plant growth by modifying root morphology by producing plant hormones and other growth-promoting factors beneficial to the host. Subsequent studies on ANF in cereal crops have identified additional N-fixing strains, including *Pseudomonas*, *Klebsiella*, and *Enterobacter*. SynCom-based inoculants can significantly enhance ANF in rice. For instance, a SynCom comprised of *Azospirillum* spp. NL1, *Bradyrhizobium* spp. NL2, and *Azotobacter* spp. NL3 increased root N-fixing activity (%Ndfa) by approximately 52%, and promoted root colonization and plant architecture (shoot ramification and tillering) (Tran et al., 2024). Therefore, SynComs are ecological tools and functional biofertilizers in cereals crops. Table 1 summarizes the diversity and functional roles of associative N-fixing bacteria.

**Table 1:** Impact of associative nitrogen-fixing bacteria in major Poaceae crops and their beneficial responses.

Crop	Bacterium/Group	Experimental condition	Functional mechanism	Impact	Plant N gain	Source
Maize (*Zea mays* L.)	*Azospirillum brasilense*	Greenhouse and field	Auxin and cytokinin production, root architecture modification	Increases yield, biomass, and iron uptake	No	Housh et al. (2021); Hungria et al. (2022)
	*Herbaspirillum seropedicae*	Greenhouse and field	Endophytic colonization, *nif* gene expression, phytohormone production, in planta N fixation	Promotes maize growth through BNF	Indirect	Waller et al. (2021)
	*Kosakonia sacchari* (engineered)	Laboratory and greenhouse	*Nif* regulon regulation/genetic rewiring (GlnD–PII signaling described in optimization work)	Maintains N fixation under high N inputs	No	Bloch et al. (2020)
	*Pseudomonas stutzeri* (engineered)	Greenhouse and field	Nitrogen fixation and engineered ammonium excretion; IAA production; PGP traits	Increased maize growth and grain yield by ~5–6%.	Yes (NH₄^+^ release measured)	Jiang et al. (2022)
	Mucilage-associated microbiota (Sierra Mixe landrace)	Field	Low-O₂ mucilage niche supporting diazotrophic community; high nitrogenase activity	29–82% of plant N from the atmospheric (%Ndfa)	Yes (direct %Ndfa)	Van Deynze et al. (2018)
	Maize landraces with aerial roots and mucilage	Field	Aerial root-mediated BNF through mucilage-associated diazotrophs	Significant variation in %Ndfa; some comparable to *Sierra Mixe*	Yes (direct %Ndfa)	Connolly et al. (2025)
	Alfalfa-maize intercropping (diazotrophic community)	Field	Intercropping-driven shifts in diazotroph diversity and network complexity	Altered diazotroph community structure	No	Zhang J. et al. (2024)
	*Azotobacter* spp.	Field	Modulation of N assimilation pathways	Improved photosynthetic efficiency and salinity resilience capacity	No	Nida et al. (2024)
	Engineered/remodeled soil diazotroph community	Field	Enhanced community level of biological N fixation	Maintained productivity with partial synthetic N replacement	Indirect	Martinez-Feria et al. (2024)
	Diazotrophic inoculants (two species consortium)	Greenhouse	Root development stimulation; improved N acquisition	Increased plant N uptake and growth	Indirect (N uptake proxy)	Carvalho et al. (2023)
Wheat (*Triticum aestivum* L.)	*Azospirillum brasilense*	Greenhouse and field	Hormone induction, root trait enhancement; improved N metabolism	Increased yield and N-use efficiency	Indirect (N-use efficiency proxy)	Galindo et al. (2022); Zaheer et al. (2022)
	*Paenibacillus beijingensis* (BJ-18)	Laboratory	N fixation; phosphate solubilization; endophytic colonization	Increased N and P content, nitrogenase activity, and biomass	Yes	Li et al. (2019); Li et al. (2020)
	*Brucella oryzae* + *B. ciceri* (B1 + B2)	Greenhouse	Synergistic N fixation; IAA production; improved soil C/N cycling	Strongly growth promotion; up to 25% N fertilizer savings	Yes	Saniya et al. (2024)
	*Azospirillum brasilense*, *Bacillus subtilis*, *Pseudomonas fluorescens*	Field	Combined N₂ fixation; phytohormone production; Zn solubilization	Improved growth, enhanced Zn concentration in leaf (38.8–45.9%), shoot (25–31%), grain (33–34%); enhanced grain yield (~20%) and Zn use efficiency	Indirect (yield/Zn proxy)	Jalal et al. (2022)
	*Spirillum* spp. (historical/root-interaction group; related to *Azospirillum*)	Laboratory	Root restructuring; modulation of nitrate transporters (NAXT/NRT family genes)	Up-regulated nitrate transporter/related genes in dual RNA-seq	No	Camilios-Neto et al. (2014); Sigalas et al. (2023)
	*Azospirillum baldaniorum/A. brasilense*	Greenhouse	Ammonia scavenging; N budget improvement	Improved seedling N status and early vigor	Indirect (seedling N status)	Dias et al. (2024)
	Free-living N-fixers + biostimulant	Field	BNF synergy with commercial bio-stimulant	Enhanced growth potential under farm conditions	No	Buisset et al. (2025)
	Salt-tolerant diazotrophs (PGP)	Greenhouse	N fixation under saline/alkaline stress	Promotes growth in techno soils; resilience to salinity	No	Kumar et al. (2024)
	BNF consortium (varied N and P regimes)	Field	Biological N₂ fixation under contrasting nutrient regimes	Agronomic advantage across nutrient regimes		Aasfar et al. (2024)
	Endophytic *Rahnella* spp.	Laboratory	Endophytic N fixation; PGP traits	Promotes crop growth and development	No	Rana et al. (2024)
	*Azospirillum brasilense* (PGPR)	Field	N uptake enhancement; agronomic efficiency; biomass accumulation	10.3% yield increase, 10.5% profit gain with reduced N	Indirect (N uptake proxy)	Galindo et al. (2022)
	Rhizobia strains (*Ensifer meliloti*, *E. mexicanus*, *Rhizobium tropici*) used as biofertilizer	Laboratory	BNF and growth promotion	Stimulates growth under controlled settings	No	Serazetdinova et al. (2025)
	Rhizosphere microbiome (community level)	Field	Fertilizer-driven shifts in N-cycling community functions	Optimized fertilization favors beneficial N functions	No	Cui et al. (2024)
	*Stenotrophomonas* sp. + NH₄^+^-excreting mutant	Laboratory and greenhouse	BNF with extracellular ammonium release	Direct N supply and improved plant growth	Yes	Zhou et al. (2025)
Rice (*Oryza sativa* L.)	*Herbaspirillum seropedicae*	Greenhouse	N₂ fixation, repression of defense genes, Fe and N uptake modulation	Enhanced plant growth, Fe and N acquisition	Yes (indirect Fe/N gene expression)	Brusamarello-Santos et al. (2019)
	*Methylobacterium* spp. (phyllosphere)	Greenhouse	Phyllosphere colonization, plant hormone modulation, and photosynthetic enhancement	Variable effects on rice, some strains improved growth and grain yield, benefits dependent on host–strain dependent	No	Sanjenbam et al. (2022)
	*Azospirillum* spp.	Field	N₂ fixation, rhizosphere colonization, phytohormone (IAA, cytokinin) production, tillering stimulation	Enhanced growth, yield and soil fertility	Indirect	Sarkodee-Addo et al. (2021)
	*Clostridium* spp. (anaerobic rhizosphere)	Field	Anaerobic heterotrophic BNF in flooded paddy soils	Contribute to positive N balance in flooded conditions	Yes	Xiao et al. (2022)
	*Methylobacterium oryzae* CBMB20	Greenhouse	Proteomic reprogramming, photosynthesis, and metabolism enhancement	Improved growth and development via genotype-specific proteomic shifts	No	Walitang et al. (2023)
	*Herbaspirillum* R3 strain (N-fixing bacterium)	Field	Rhizosphere N enrichment, *nif* gene community stimulation, N-uptake gene up-regulation	~8.8% theoretical yield increase	Indirect	Li et al. (2024)
	*Rhodobacter sphaeroides*	Field	Phototrophic N fixation in saline acid sulfate soil	Improved soil–plant N and rice performance over two seasons	Yes	Anh et al. (2025)
	Engineered *Rhodopseudomonas palustris* (Fe-only nitrogenase, NifA* regulated)	Greenhouse	Constitutive Fe-only nitrogenase expression under NH₄^+^	Stimulated rice growth	Indirect	Zeng et al. (2024)
	Terrestrial N-fixing cyanobacteria	Field	Cyanobacterial N₂ fixation	Promoted rice growth	Indirect	Wu et al. (2024)
Sugarcane (*Saccharum officinarum* L.)	*Klebsiella variicola* (DX120E)	Greenhouse	Endophytic colonization influences polyamine metabolism and PGP hormone pathways	Improved growth via hormone and polyamine modulation	No	Qin et al. (2024)
	*Enterobacter roggenkampii* (ED5)	Greenhouse	GS/GDH and ammonium-assimilation pathway effects; N-metabolism enzyme modulation	Improved N metabolism under varying N regimes	Indirect (enzyme activity)	Guo et al. (2023b)
	*Acidithiobacillus* spp. + *Beijerinckia indica*	Field	Rock phosphate solubilization, N fixation, organic matter decomposition, and nutrient release	Improved nutrient uptake, enhanced soil fertility, comparable to soluble fertilizers	Indirect	Nunes Oliveira et al. (2017)
	*Klebsiella variicola* DX120E	Greenhouse	N₂-fixation, enhanced C and N metabolism, IAA, phosphate solubilization, siderophore production	Boosted physiological traits and yield related responses	Yes	Qin et al. (2022)
	*Azospirillum brasilense* inoculation	Greenhouse	N₂-fixation, phytohormone production, phosphate solubilization	Increase aboveground fresh mass and dry mass	Indirect	de Souza et al. (2025)
	*Bacillus megaterium* (CY5) and *Bacillus mycoides* (CA1)	Laboratory and greenhouse	*nifH* expression, biological N fixation	Improved germination, biomass accumulation, and boost nitrogenase activity	Indirect	Singh et al. (2020)
	*Pseudomonas* (diazotrophic, PGP)	Greenhouse	N_2_-fixation with phytohormone stimulation	Growth promotion, potential N input reduction	No	Singh et al. (2024)
	Diazotrophic inoculants (mixed)	Field	Enhanced soil N and BNF contribution	Higher effective N supply	Yes	Martins et al. (2020)
	Endophytes: *Pantoea cypripedii* AF1; *Kosakonia arachidis* EF1	Greenhouse	Endophytic N assimilation and PGP traits	Enhanced N assimilation and plant growth	Indirect	Singh et al. (2021)

## 3 Plant compartments of associative nitrogen fixation in Poaceae crops

In cereal crops, associative N-fixing bacteria localize to the rhizosphere, roots, and leaves, with the rhizosphere serving as the primary site for the colonization and activity of N-fixing microorganisms. The rhizosphere provides abundant organic matter and a favorable micro-environment, which supports the growth of N-fixing microorganisms and enhances N fixation. Moreover, the roots of cereal crops secrete organic compounds, including sugars, amino acids, and organic acids, which provide carbon sources and energy for N-fixing microorganisms, promoting their colonization in the rhizosphere. For instance, the flavonoid naringenin significantly enhanced the colonization of *Azospirillum caulinodans* in the wheat rhizosphere (Webster et al., 1998). The mucilage secreted by maize aerial roots recruits N-fixing bacteria, creating favorable conditions for bacterial growth, while the N-fixing bacteria supply the required N for the growth of host plant phenomenon observed in various plant species (Van Deynze et al., 2018; Carter et al., 2019; Sinha Roy et al., 2002; Pang et al., 2023). The interactive mechanism may be a common feature of BNF in plant roots. Enhancing apigenin secretion in plant roots increases yield under N-deficient conditions in rice (Yan et al., 2022). The bacterial biofilm, composed of polysaccharides, proteins, and lipids, influences the interaction between N-fixing bacteria and plant roots (Flemming et al., 2016; Wang D. et al., 2017; Nag et al., 2020). In some cases, biofilm formation is essential for successful root colonization (Meneses et al., 2011; Flemming et al., 2016). The rhizosphere not only hosts a wide variety of N-fixing microorganisms but also includes other symbiotic microbes that collectively contribute to the nitrogen cycle and nutrient uptake in crops.

The discovery of endophytic N-fixing bacteria has significantly altered the understanding of N fixation in non-leguminous crops. Specific N-fixing microorganisms can colonize the roots or root cortex of cereal crops, and performing endophytic N fixation. Endophytic N fixation differs from rhizosphere N fixation, where microorganisms penetrate the root or root cortex cells of Poaceae crops, establishing a mutualistic symbiosis with the plant through micropores or intercellular spaces. Once inside the plant, these microorganisms primarily colonize root cortical cells, mesophyll, parenchyma, and xylem vessels. For example, *Herbaspirillum seropedicae* Z67 enters the root through cracks at lateral root emergence sites, colonizing the intercellular spaces within the roots (James et al., 2002). In maize, rice, and wheat, *Gluconacetobacter diazotrophicus* colonized the root cell cytoplasm, where conditions favor the expression of nitrogenase genes (Cocking et al., 2006).

However, N-fixing endophytic bacteria face major challenge as a entering and surviving in the plant. Thus, these bacteria have evolved various traits to overcome this challenge. For instance, some bacteria possess genes associated with chemotaxis, flagella, and pili formation, as well as the recognition and degradation of plant-derived polysaccharides (Fouts et al., 2008). Some bacteria have evolved traits that enhance their ability to penetrate plant tissues. For instance, the endophytic N-fixing bacterium *Azoarcus* sp. BH72 possesses genes that encode cell-wall degrading enzymes (CWDEs), such as cellulases and polygalacturonases (Krause et al., 2006). Additionally, during the initial stage of endophytic colonization, plant defense responses are activated, releasing reactive oxygen species (ROS) within cells, which imposes osmotic stress on the endophytic bacteria. Thus, mitigating ROS-induced damage is crucial for enhancing colonization. In the case of *Gluconacetobacter diazotrophicus* PAL5, endophytic colonization significantly up-regulated the transcription levels of ROS-detoxifying genes, such as superoxide dismutase (SOD) and glutathione reductase (GR), indicating that the ROS-scavenging enzyme system of strain PAL5 is vital in the colonization of rice (Alquéres et al., 2013).

Although roots are the primary site of N fixation, N-fixing microorganisms can also colonize and fix N in the leaves and other aerial organs of Poaceae crops since the vascular bundles in the crop stems provide colonization sites for N-fixing bacteria. The pore of the perforation plates between xylem elements facilitates the smooth passage of bacteria (Bové and Garnier, 2003). For example, after colonizing the intercellular spaces of root tissues, the N-fixing bacterium *Herbaspirillum seropedicae* Z67 can penetrate the endodermis, enter the root xylem vessels, and eventually colonize the intercellular spaces of stem and leaf epidermal cells in the substomatal cavities (James et al., 2002). In maize, Zhang et al. (2022) showed the number of N-cycle-related microorganisms was significantly higher in the xylem sap of the stem than other plant parts. Moreover, a species of diazotrophic *Gluconacetobacter* has been identified in the stems and leaves of sugarcane (Cavalcante and Dobereiner, 1988). The plant phyllosphere, which includes the episphere (epiphytic layer) and endosphere (endophytic layer), also provides an important habitat for N-fixing bacteria (Zhu et al., 2023). For instance, the N-fixing bacteria in the maize phyllosphere predominantly belong to the *Enterobacter* genus (Abadi et al., 2021), whereas those in the rice phyllosphere are mainly from the *Acinetobacter* genus (Knief et al., 2012). However, the α-diversity of N-fixing microbial communities in the epiphytic layer of the phyllosphere is significantly higher than endophytic layer (Zhu et al., 2023).

## 4 Mechanisms of associative nitrogen fixation in Poaceae crops

Deciphering the mechanisms of ANF in maize, wheat, and rice plants provides valuable insights for optimizing N fixation strategies in sugarcane. *Pseudomonas* A1501 synergistically enhances N fixation and hormone regulation in maize, increasing plant biomass by 25.4% (Ke et al., 2019). Similarly, *Kosakonia sacchari PBC6.1,* isolated from maize roots, dynamically modulates low-N responses via the GlnD-PII signaling axis. The optimal abundance of *nifH* genes in xylem-associated γ-Proteobacteria indicates the presence of diazotrophic communities within plant vascular tissues, suggesting a potential capacity for *in planta* nitrogen fixation. However, *nifH* gene abundance alone does not confirm active fixation or the transfer of fixed nitrogen to the plant; isotopic tracing or direct quantification of plant N derived from atmospheric N_2_ (%Ndfa) (Wang et al., 2021; Guo K. et al., 2023). Wheat plants and soil microbes share a close functional relationship in which bacteria, such as *Bacillus* can enhance N-use efficiency by up-regulating plant N metabolism genes, including glutamine synthetase (Li et al., 2019; Chen et al., 2022). It should be noted, however, that improved NUE reflects more efficient assimilation of existing N sources and interpreted as evidence of fixed-nitrogen transfer from the bacterium to the plant. Nitrogen-fixing *Spirillum* bacteria can alter plant root morphology by activating the nitrate transporter gene (*NAXT*), stimulating soil nitrate uptake and generating a feedback loop of microbial colonization and enhanced plant N acquisition (Camilios-Neto et al., 2014). This mechanism reflects diazotroph-mediated enhancement of plant nitrogen uptake capacity rather than the direct delivery of bacterially fixed nitrogen. Specific strains of plant growth-promoting rhizobia can colonize non-legumes hosts, such as rice, improving single-leaf net photosynthetic rate and increasing total biomass or grain yield by 10–43%, depending on strain and growth conditions. Slow-growing rhizobia colonize the phyllosphere (aerial leaf surfaces) of rice have been shown to significantly enhance photosynthetic efficiency, increasing yield by 1,000 kg/hm^2^ (Padukkage et al., 2021; Ofori et al., 2024). Furthermore, *Pseudomonas*-*Rhodotorula* microbial consortia rapidly induce root nodule gene expression within 24 h, revealing the potential of across-kingdom microbial synergy to support plant growth and development (Paul et al., 2020).

Sugarcane ANF system offers a broader perspective characterized by more complex and multidimensional regulatory features, demonstrating how diverse microbial consortia and host regulatory pathways converge to enhance N fixation efficiency. The endophytic bacterium *Enterobacter roggenkampii* ED5 in sugarcane has been shown to direct ammonium into plant glutamine synthesis via the GS/GDH dual pathway (Song et al., 2022). While ammonium is a product of nitrogenase activity, this biochemical process represents a potential pathway for fixed-N delivery to the host; isotopic labelling (^15^N_2_) would be required to confirm that the ammonium assimilated by the plant is derived from atmospheric N_2_ fixation rather than from other N sources. *Klebsiella variicola* enhances polyamine metabolism and elevates auxin levels by 37.4%, demonstrating a spatiotemporal coordination between bacterial metabolic activity and host developmental regulation (Guo et al., 2023a, 2023b; Qin et al., 2024). These effects are attributed to hormone-mediated plant growth promotion; the contribution of bacterially fixed nitrogen to plant N status was not directly quantified in this study.

*Pseudomonas aeruginosa* DJ06 carries 12 plant-growth-promoting gene clusters including that encoding siderophore biosynthesis (ent) and phosphate metabolism (pho) conferring broad-spectrum nutrient regulation capabilities. The biofilm formation capacity enhances colonization stability by 3.8-fold relative to *Bacillus*-like bacteria in wheat. In sugarcane, the induction of auxin signaling increases sugarcane lateral root density by 62%, establishing a tripartite functional module encompassing N fixation, phosphorus mobilization, and disease resistance (Guo et al., 2023b). Therefore, future research should integrate maize’s cross-organ microbiome localization techniques, rice’s phyllosphere nitrogen fixation enhancement strategies, and sugarcane’s multi-gene-cluster engineering approaches to establish a universal theoretical framework for associative nitrogen fixation. Critically, progress in this field requires moving beyond proxy indicators of diazotrophic potential, such as *nifH* expression, acetylene reduction activity, and ammonium release toward direct quantification of plant N derived from atmospheric N_2_ using ^15^N-based methods, which remains the accepted standard for demonstrating genuine fixed-nitrogen transfer to the plants.

## 5 Regulative mechanisms of associative nitrogen fixation

### 5.1 Plant regulation of nitrogen fixing bacteria

The relationship between cereal crops and nitrogen-fixing bacteria is highly complex, with nitrogenase serving as the central enzyme responsible for converting atmospheric N_2_ into NH_3_, a form readily absorbable by plant roots (Rutledge et al., 2022). As the essential catalyst of biological nitrogen fixation, nitrogenase requires 16 ATP molecules and 8 electrons to reduce one molecule of N_2_ into two molecules of NH_3_ and one molecule of H_2_, reflecting the considerable biochemical process. Consequently, optimizing nitrogenase activity is a key strategy for enhancing overall nitrogen fixation efficiency; however, ANF remains constrained by several critical limiting factors, including oxygen-induced inhibition of the nitrogenase complex, nitrogen-dependent repression of nitrogenase gene expression, and the substantial energetic demands of the fixation reaction (Kuypers et al., 2018). Nitrogenase is only active under low-oxygen conditions, such as the unique N-fixing bacterium, *Gluconacetobacter diazotrophicus,* which reduces the surrounding oxygen concentration of sugarcane by enhancing respiration. This deoxygenation protects the temporarily inactivated nitrogenase from oxidative damage by forming a complex with the FeSII protein (Ureta and Nordlund, 2002). Additionally, this bacterium secretes levansucrase, which hydrolyzes sucrose into glucose and fructose while catalyzing levan synthesis and forming an extracellular polysaccharide barrier that restricts oxygen diffusion and protects nitrogenase (Dong et al., 2002; Velázquez-Hernández et al., 2011; Yu G.-H. et al., 2021).

Host plants actively regulate N fixation by secreting flavonoids, coumarins, and organic acids to attract and activate N-fixing bacteria (Liu et al., 2022). In wheat, naringenin and soyasaponins facilitate the colonization of the endophytic N-fixing bacterium *Azorhizobium caulinodans* ORS571 mitigating ammonia stress (Gough et al., 1997). In legume-cereal intercropping systems, root exudates from cereal plants can induce key genes that promote nodule formation in N-fixing bacteria and enhance N fixation capacity. The addition of maize root exudates in the rhizosphere of faba bean can activate genes related to nodulation factor synthesis, rapidly triggering nodulation under low N condition. The key nodulation genes remain up-regulated, promoting N fixation.

Root exudates from cereal crops indirectly enhance the N-fixing bacterial activity by modulating the structure of the rhizosphere microbial community (Jauregui et al., 2023; Liu et al., 2024), thus suppressing the growth of non-N-fixing bacteria. In contrast, the roots of cereal crops directly and indirectly enhance N fixation by secreting hormones, such as indole-3-acetic acid (IAA), which promote root growth, nutrient uptake, and the expression of N-fixing bacterial genes (Ali et al., 2021). Moreover, this interaction may establish a positive feedback loop since the ammonia fixed by N-fixing bacteria absorbed and assimilated by the plants, which influences the microbial community and promotes N fixation.

The host plant genotype also influences the selection of N-fixing bacterial communities. For example, *Methylobacterium* is more abundant in the rhizosphere of wild rice than cultivated rice, suggesting that different rice varieties genetically regulate the recruitment of beneficial N-fixing bacteria (Yin et al., 2023). Host plants genetically regulate nutrient stress by integrating stress signals and recruiting more functional microorganisms associated with N acquisition (Yu P. et al., 2021; Meier et al., 2022; Zhang L. et al., 2024). Thus, the symbiotic mechanisms between legumes and rhizobia are clear (Luo et al., 2023), but the signaling pathways and regulatory mechanisms between cereal crops and N-fixing microorganisms remain unclear. Future research should focus on the complex interactions between plant genotypes, host phenotypes, and N-fixing bacterial communities to provide theoretical and technical support for enhancing ANF efficiency.

### 5.2 Synergistic regulation between microorganisms

Interactions among microorganisms are pivotal in regulating the functional potential of N-fixing microbes, forming a complex network of interactions between N-fixing bacteria and other microorganisms, including bacteria, fungi, and viruses. Zhang et al. (2022) identified two N-fixing bacteria and twelve non-N-fixing bacteria in the xylem sap of maize stems, with the latter differentially enhancing nitrogenase activity in N-fixing bacteria. These non-N-fixing bacteria may assist N-fixing bacteria in N fixation by modulating N and oxygen concentrations in the xylem micro-environment (Yu H. et al., 2021; Xiao et al., 2023). Moreover, multiple microbial network studies have demonstrated that the synergistic interaction between arbuscular mycorrhizal fungi and N-fixing bacteria significantly enhances N fixation efficiency (Luo et al., 2023; Sun et al., 2015).

Under low-nutrient conditions, rice and barley plants may perceive LCO and CO signals, indicating that the interaction mechanism may function in non-leguminous crops. However, the specific mechanisms by which fungi and their secreted signals regulate N fixation in cereal crops require further investigation (Li et al., 2022). SynComs serve as experimental testbeds to unpack the molecular dialogues underpinning plant–microbe and microbe–microbe communication (Fonseca-García et al., 2024). Thus, it provides insights into cooperative behavior, such as nutrient exchange and signaling, which are pivotal in regulating N-fixation synergism. Viral communities play a critical role in shaping microbial community structures and transferring auxiliary metabolic genes, thereby participating in the carbon and N cycles (Lopez-Simon et al., 2023; Tong et al., 2023). Under N-limited conditions, bacteriophage-resistant strains exhibit significant nutrient deficiencies than wild-type cyanobacteria, suggesting that bacteriophage resistance may constrain the N fixation capacity (Kolan et al., 2024).

### 5.3 Regulation of nitrogen-fixing microorganisms by abiotic factors

Oxygen and N are the key factors influencing BNF by directly inhibiting nitrogenase activity, and N fixation process is highly energy-intensive. Nitrogenase is highly sensitive to oxygen and can only maintain its normal function under aerobic conditions (Kuypers et al., 2018). Thus, the significantly higher N fixation efficiency in the paddy soil of eastern China than dry-land soil is possibly due to the fluctuations in oxygen concentration caused by intermittent irrigation and prolonged flooding (Li et al., 2023). Furthermore, BNF is an energy-intensive process, requiring 8 high-energy electrons and 16 ATP molecules per nitrogen molecule fixed. In cereal crops, the symbiotic relationship with nitrogen-fixing bacteria is relatively loose, unlike the root nodules in legumes that provide a continuous energy supply to nitrogen-fixing bacteria. As a result, nitrogen-fixing bacteria in cereal crops tend to absorb nitrogen nutrients directly from the soil, which contributes to their lower N fixation efficiency.

The long-term use of inorganic N fertilizers in agricultural practices further suppresses the N-fixing function of N-fixing bacteria, reducing the abundance and diversity of N-fixation (*nif*) genes in microbial communities (Fan et al., 2019; Chen et al., 2021; Norman and Friesen, 2017). For example, 35 years of inorganic N fertilizer application reduced (50%) the soil N fixation efficiency in wheat-soybean rotation fields. Similarly, continuous N fertilizer application in maize fields over five years resulted in ~54–80% reduction in the abundance of *nifH* genes in the soil (Fan et al., 2019; Chen et al., 2021). In contrast, applying organic fertilizers, such as manure, biochar, and straw, generally enhances BNF efficiency, likely due to improvements in soil organic carbon, iron minerals, pH, and microbial community structure (Shi et al., 2021; Yu H. et al., 2021). A global meta-analysis has shown that increasing the soil organic carbon content can mitigate the inhibitory effect of N on N fixation (Zheng et al., 2023). Yu H. et al., (2021) showed that the long-term application of organic fertilizers significantly increased the short-range ordered iron minerals in the soil, raising the total Mo content by 30%, increasing the *nifH* gene abundance by 14%, and enhancing the nitrogenase activity (60%). Organic fertilizers mitigated the inhibitory effect of low pH on nitrogenase (Shi et al., 2021; Yu P. et al., 2021). Furthermore, diurnal temperature fluctuations significantly affect the N fixation process, such as the *Escherichia coli* and *Klebsiella oxytoca,* which release fixed N extracellularly at night (23 °C) in a form available for plant uptake, offering new insights into N fixation regulation (Tang et al., 2023). Overall, oxygen concentration, N fertilizer application, and temperature collectively form a complex regulatory network that directly influences nitrogenase activity and N fixation efficiency.

## 6 Main ways to improve the efficiency of nitrogen fixation

### 6.1 Engineering modification of nitrogen-fixing microorganisms

Given the technical complexity of plant N fixation engineering, modifying plant-associated microbiomes has emerged as a viable alternative. Engineering N-fixing bacteria can enhance N fixation efficiency in non-leguminous crops and confer N-fixing ability to non-N-fixing bacteria, thereby compensating for the limited presence of native N-fixing bacteria in the rhizosphere (Wang et al., 2013), demonstrating that only a few genetic elements are required for heterologous N fixation. However, nitrogenase activity is generally lower in heterologous hosts than native N-fixing bacteria, highlighting the need for optimizing gene clusters through synthetic biology (Li et al., 2016; Bloch et al., 2020). This approach has been successfully applied to N-fixing bacteria, such as *Klebsiella oxytoca* and *Paenibacillus*, providing a solid theoretical foundation for bacterial engineering interventions (Li et al., 2016; Smanski et al., 2014). Furthermore, external N levels during the N fixation process strongly influence N-fixing bacteria. These bacteria typically cease N fixation to conserve energy when N is abundant. Therefore, it is essential to modify N-fixing bacteria to avoid disrupting their N fixation process by external N concentrations, thus ensuring the continuous and efficient production of plant-available N. The expression of *nif* is primarily regulated by two proteins, i.e., the *NifA* activator, which promotes *nif* gene transcription, and the *NifL* repressor, which inhibits *NifA* activity under aerobic or excess N conditions (Ambrosio et al., 2017). Modifying these two genes can enable the engineered bacteria to sustain N fixation under abundant external N and ammonium absorption via the ammonium transporter AmtB. However, disrupting AmtB can prevent ammonium uptake, releasing fixed ammonium into the rhizosphere for plant use (Zhang et al., 2012). Similarly, engineering strategies that enhance ammonium export can further improve the N fixation efficiency (Pankievicz et al., 2019). Nitrogenase reduces atmospheric N to biologically available ammonia (NH₃), but it is highly sensitive to readily inactivated by oxygen. Thus, N-fixing microorganisms overcome this limitation through various protective mechanisms, especially the small iron–sulfur cluster protein, Shethna protein II (*FeSII*), to protect nitrogenase through conformational changes (Narehood et al., 2025). Introducing oxygen-protective proteins (e.g., Shethna protein) into nitrogen-fixing bacteria may effectively mitigate the inhibitory effects of oxygen on nitrogenase, thereby enhancing nitrogen fixation activity.

The colonization ability of nitrogen-fixing bacteria in host roots is crucial. Bacterial chemotaxis, a key feature of the two-component signaling system, plays a vital role in guiding bacterial movement. Histidine kinase CheA undergoes autophosphorylation and transfers a high-energy phosphate group to CheY, which regulates flagellar rotation and influences bacterial swimming patterns. In maize, root exudates significantly upregulate the expression of the *cheA* gene in the degradative bacterium *Hansschlegelia zhihuaiae* S113, promoting its aggregation in the rhizosphere and facilitating colonization (Zhang et al., 2021). Additionally, extracellular polysaccharides (EPS) produced by N-fixing bacteria protect cells from oxidative stress in *in vitro* and during root colonization in rice (Meneses et al., 2017). Engineering modifications to chemotaxis-related genes (e.g., *cheA*) or biofilm formation genes (e.g., *eps*) can further enhance the adhesion and colonization ability of nitrogen-fixing bacteria in plant roots.

### 6.2 Engineering nitrogen fixation process

Nitrogen fixation system into Poaceae crops presents a promising strategy for enhancing ANF. However, the heterologous expression of the nitrogenase system hinders autonomous N fixation in these crops. The oxygen-sensitive metalloprotein complex, nitrogenase function requires overcoming key obstacles, such as maintaining enzyme complex stability, balancing oxygen sensitivity with energy metabolism, and precisely coordinating the expression of multiple genes. Thus, researchers have explored sub-cellular localization, genetic engineering optimization, and modifications to symbiotic signaling pathways. The oxygen sensitivity of nitrogenase makes the mitochondria and chloroplasts, both relatively low-oxygen environments, ideal sites for its localization (Soto et al., 2013; Bennett et al., 2020). Mitochondrial genome editing is theoretically feasible, but its low transformation efficiency limits application. Consequently, the prevailing strategy is nuclear genome integration followed by targeted peptide-mediated transport. For example, Burén et al. (2017) achieved mitochondrial targeting by integrating nine *nif* genes into the yeast nuclear genome using the SU9 sequence. Allen et al. (2017) employed the pFAγ leader sequence to direct 16 *Nif* proteins, demonstrating the feasibility of cross-species transport. However, the key component *NifD* is unstable, and its degradation is linked to R98/Y100 residues and enzyme activity processing.

Introducing R98P/Y100Q mutations significantly enhances *NifD* stability (Xiang et al., 2020). Although the limited cluster synthesis capacity of plant mitochondria [Fe-S] restricts *NifH* functionality. This bottleneck can be overcome using thermophilic bacterial-derived *NifH* alongside *NifU* protein supplementation (Jiang et al., 2021). The plastid system has also garnered attention due to its advantage in polycistronic expression. Ivleva et al. (2016) achieved limited activity when expressing *nifH/M* in tobacco plastids. Nonetheless, Eseverri et al. (2020) successfully reconstructed the *nif* gene cluster from *Azotobacter vinelandii* using synthetic biology and chloroplast-targeting system, achieving high *NifH* activity. Spatiotemporal regulation strategies, such as hypoxia-inducible promoters, can mitigate the conflict between N fixation and photosynthetic oxygen production (Bothe et al., 2010). Additionally, donor strain selection and gene module simplification, such as the minimal expression of the *Bacillus nif* gene cluster in *Arabidopsis,* have improved system compatibility (Burén et al., 2017; Yao et al., 2021). Nitrogenase functionality also depends on precise gene expression ratios (Temme et al., 2012) and synthetic biology offers innovative approaches to address this challenge. Wang et al. (2013) and Yang et al. (2014, 2018) reduced regulatory complexity via bypassing Mo cofactor dependence through gene fusion and polyprotein strategies. In transgenic *Arabidopsis*, an optimized *nif* gene cluster exhibited high nitrogenase activity and increased biomass under low-N conditions. Li et al. (2016), and Yao et al. (2021) confirming the feasibility of this approach. However, epigenetic silencing caused by multiple gene insertions remains a significant challenge, underscoring the need for novel anti-silencing expression frameworks or inducible regulatory elements.

Rhizosphere metabolite engineering provides a novel strategy for enhancing ANF. For instance, knocking out *CYP75B3/4* in rice increased the secretion of apigenin, significantly promoting N-fixing bacterial colonization (Yan et al., 2022). Similarly, transgenic barley synthesizing rhizopine specifically induced nitrogenase expression (Haskett et al., 2022). Future research requires a systemic investigation of additional metabolites to enhance N-fixing bacterial function and specific metabolites that selectively interact with specific N-fixing bacteria.

### 6.3 Plant-mediated approaches for microbiome engineering

Plant-mediated approaches for positioning grasses-associated microbiomes, particularly diazotrophic bacteria are a rapidly growing field in sustainable agriculture. Thus, functional genomics, molecular biology and plant breeding are converging to allow targeted recruitment, enrichment, and engineering of beneficial microbial consortia. Figure 2 presents an overview of integrated strategies for engineering diazotrophs and plant-associated microbiomes. These approaches allow breeders to enhance N fixation, nutrient uptake, and stress resilience in major Poaceae crops. Overuse of synthetic N fertilizers undesirably impacts the environment, increases contamination, and damages natural resources. Thus, plant-mediated approaches offer a combination of genome-wide association studies (GWAS), RNA interference (RNAi), CRISPR/Cas genome editing, cytological visualization, and selective breeding to optimize or manipulate beneficial microbial consortia.

**Figure 2:**
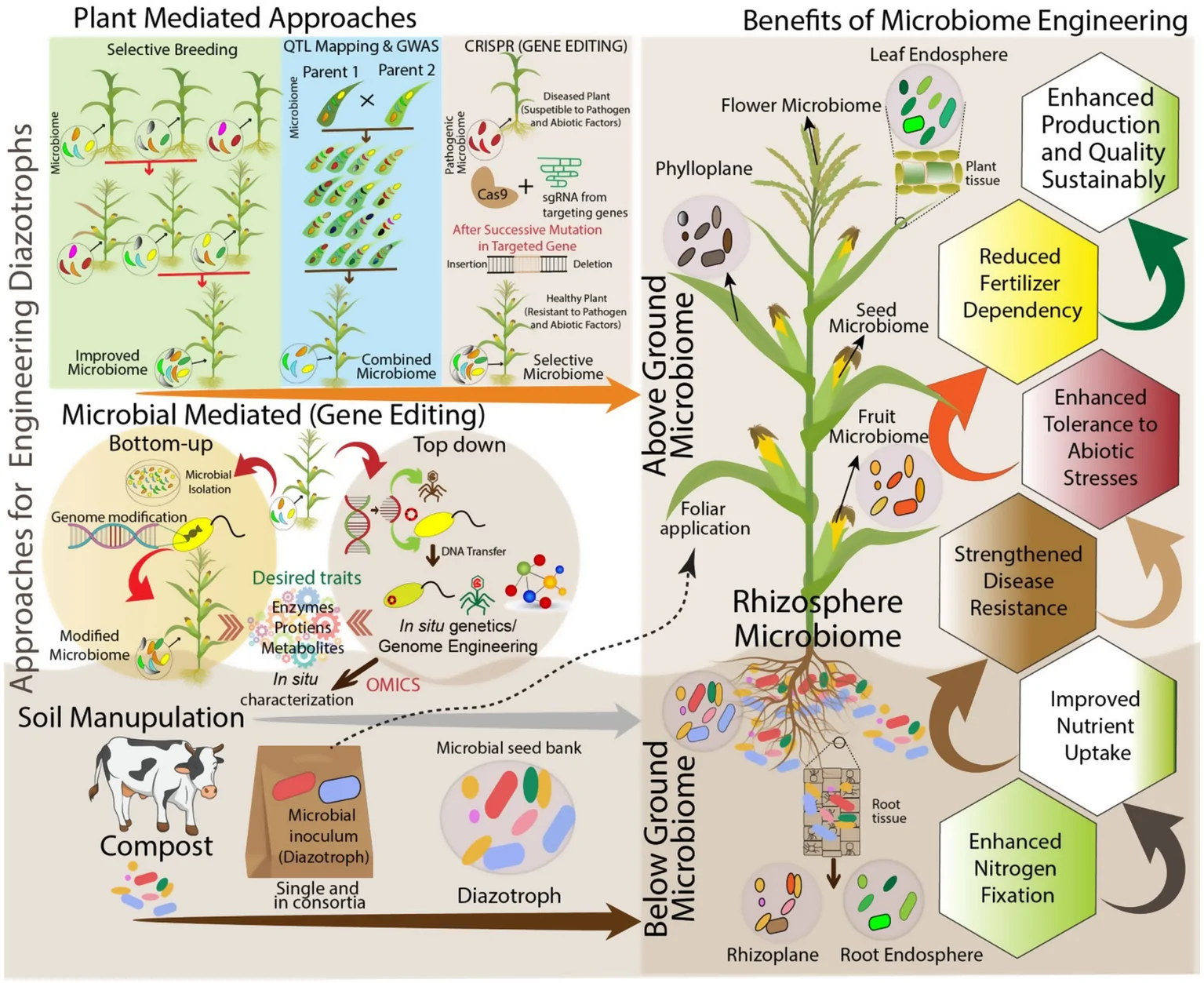
Integrated strategies for engineering plant-associated microbiomes to improve biological N fixation and crop performance. The methods include selective breeding, genome-wide association studies (GWAS), CRISPR editing, soil manipulation, and synthetic microbial consortia. QTL = quantitative trait locus.

Genome-wide association studies have been pivotal in elucidating how host genetic variation shapes the diversity and function of root-associated microbiomes. In maize, the identification of the loci linked to the abundance of N-fixing bacteria, such as *Herbaspirillum* and *Azospirillum*, reveals that allelic variation in root exudation pathways and immune signaling positively influences diazotroph recruitment and persistence in the rhizosphere and endosphere (Meier et al., 2022). Similarly, GWAS has pinpointed quantitative trait loci (QTLs) related to root architecture and metabolite secretion in rice, correlating with the enrichment of *Burkholderia* and *Gluconacetobacter* species. Hence, natural variation in secondary metabolite pathways, particularly flavonoid biosynthesis, can predictably alter diazotroph colonization patterns (Pang et al., 2023). In maize, GWAS analysis has uncovered associations between NUE traits and shifts in diazotrophic communities, suggesting that selection for high NUE genotypes has inadvertently altered the capacity of the plant to support beneficial microbes, an effect that can be harnessed in breeding programs to optimize plant–microbe interactions (Massahiro Yassue et al., 2023). These findings highlight GWAS as a critical tool for bridging host genetics and microbiome engineering to enable marker-assisted selection of genotypes that harbor efficient N-fixing microbiomes across diverse agroecosystems.

RNA interference provides a powerful reverse-genetics approach for dissecting and manipulating plant pathways that regulate diazotroph colonization and N fixation. For instance, RNAi-based suppression of defense-related receptor-like kinases in rice enhances colonization by *Herbaspirillum seropedicae*, confirming that innate immunity modulates diazotrophic symbiosis (Rosman et al., 2024). In maize, RNAi knockdown of flavonoid biosynthetic genes alters root exudate composition, reducing recruitment of *Azospirillum brasilense* and highlighting the role of targeted downregulation in rhizosphere signaling (Yan et al., 2022). In sugarcane, RNAi-mediated suppression of lignin biosynthesis genes facilitates deeper endophytic penetration by diazotrophs, enhancing nitrogenase gene expression and ammonia assimilation under low-N conditions (James and Olivares, 1998). These studies establish RNAi as a versatile tool for functional dissection and targeted modulation of plant traits that influence microbiome assembly, particularly for diazotroph plant interactions in cereal crops. Further, CRISPR/Cas genome editing has revolutionized plant-mediated microbiome manipulation by enabling precise modifications of host and microbial genomes. The CRISPR/Cas genome editing tool manipulates microbiomes by enabling particular host and microbial genome alterations. This approach enhances N fixation by upregulating genes that promote symbiotic interactions with diazotrophic bacteria, thus reducing reliance on synthetic fertilizers (Guo et al., 2023a; Basu et al., 2023). For instance, CRISPR/Cas9 interrupts apigenin catabolism genes in rice, boosting flavonoid exudates that stimulate N-fixing microbial communities in the rhizosphere (Guo et al., 2023a).

Genome editing of bacterial strains like Kv137 dissociates N fixation by allowing associative fixation in fertilized cereal crop fields without suppression (Wen et al., 2021). In maize landraces, CRISPR models highlight mucilage-secreting aerial roots as diazotroph niches, aiding host trait optimization for improved N acquisition (Van Deynze et al., 2018; Brock et al., 2025). Wider applications include engineering wheat and sorghum for microbiome resilience against abiotic stresses, accelerating trait stacking for sustainable yields (Basu et al., 2023; Hillary and Ceasar, 2019). CRISPR interference systems block essential N fixation genes in microbes, enabling adjusted expression for gramineous associations (Russell et al., 2024). These developments improve cereal crop improvement, including herbicide tolerance in maize and enhanced oil composition, indirectly benefiting the microbiome health (Hillary and Ceasar, 2019). CRISPRs also nurtures self-fertilizing crops by manipulating host exudates and microbial pathways, addressing food security to regulate climate challenges (Wang et al., 2016; Wang L. et al., 2017; Saeed, 2025). Ongoing research integrates CRISPRs with speed breeding for rapid dispersal in global sustainable agriculture (Ahmar et al., 2024).

Cytological and imaging-based approaches have provided critical insights into the spatial dynamics of diazotroph colonization, informing plant-mediated manipulation strategies. Advanced microscopy techniques, such as confocal laser scanning microscopy, fluorescence *in situ* hybridization (FISH), and fluorescent protein tagging, have revealed how *Herbaspirillum, Azospirillum,* and *Gluconacetobacter* colonize diverse niches, including the rice root cortex, maize xylem vessels, and wheat leaf intercellular spaces (James and Olivares, 1998). Cytological studies of maize aerial root mucilage have shown dense communities of N-fixing bacteria, with spatial organization maintaining microaerobic conditions essential for nitrogenase activity (Van Deynze et al., 2018). In sugarcane parenchyma cells, transmission electron microscopy (TEM) and histochemical staining have identified diazotrophs closely associated with host mitochondria and chloroplasts, suggesting metabolic cross-talk that supports energy-intensive N fixation (Cavalcante and Dobereiner, 1988). These insights highlight structural traits like root hair density, mucilage secretion, and vascular architecture as key determinants of colonization success, identifying phenotypes for breeding or engineering to enhance diazotroph recruitment (Reinhold-Hurek and Hurek, 2011). Cytological validation of CRISPRs- or RNAi-modified plants further bridges molecular interventions with ecological outcomes.

Selective breeding remains a cornerstone of crop improvement, offering a sustainable approach to enhance ANF in cereal crops. Long-term field trials in maize have shown that certain landraces preferentially recruit *Azospirillum* and *Herbaspirillum*, significantly contributing to plant N requirements (Wallace et al., 2018). In rice, traditional landraces from N-poor regions of Asia maintain higher abundances of *Burkholderia* and *Gluconacetobacter*, co-segregating with specific alleles in flavonoid biosynthesis genes (Huang et al., 2014). Wheat breeding exhibits genetic variation in recruiting N-fixing endophytes, with wild relatives harboring richer diazotrophic communities than elite cultivars (Mitter et al., 2017). Genomic selection models that treat microbial traits as heritable phenotypes, as demonstrated in maize with *nifH* gene abundance, accelerate the co-selection of beneficial microbial partnerships alongside yield and stress tolerance traits (Wallace et al., 2018).

Selective microbiome engineering complements plant-mediated approaches by directly manipulating microbial communities to favor diazotrophs. Figure 3 illustrates root-symbiotic signaling and N-acquisition mechanisms. Wheat co-inoculation with diazotrophs and arbuscular mycorrhizal fungi synergistically improves N fixation and phosphorus uptake (Mitter et al., 2017). In sugarcane, biofilm-forming *Gluconacetobacter diazotrophicus* inoculants in biodegradable carriers improve the persistence and N contribution across growth cycles (Cavalcante and Dobereiner, 1988). Microbiome transplantation from N-efficient maize landraces to elite cultivars has increased diazotroph abundance and plant growth under low-input conditions (Berendsen et al., 2012). Furthermore, SynCom strategies integrate microbial ecology with engineered design, offering a pathway to augment host–microbe compatibility and functional outcomes. Shayanthan et al. (2022) constructed SynComs tailored for agricultural contexts, emphasizing their potential to stabilize beneficial microbiome functions under diverse environmental conditions. SynComs have been engineered in rice to enhance root colonization and N-fixation activity (NL3-dominant consortia), demonstrating their feasibility as modular, high-impact biofertilizers.

**Figure 3:**
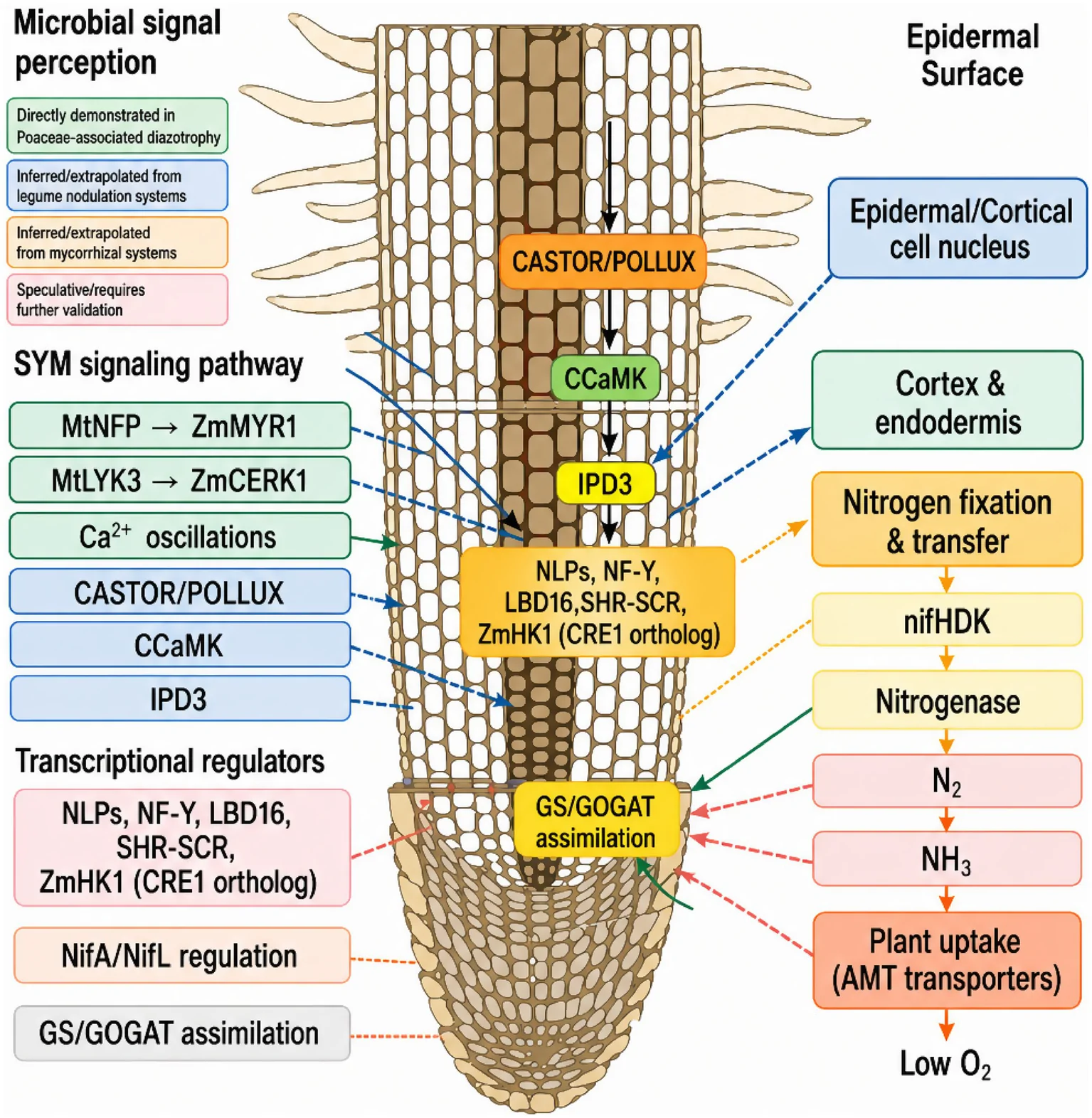
Root symbiosis and nitrogen acquisition mechanisms in Poaceae crops. Beneficial microbes trigger symbiotic signaling via receptor kinases MtLYK3 and ZmCERK1 at the epidermal surface, initiating the common symbiosis signaling pathway (CSSP) through CASTOR and POLLUX ion channels that generate Ca^2+^ oscillations decoded by Calcium–Calmodulin Dependent Protein Kinase (CCaMK) and the transcriptional regulator IPD3/CYCLOPS (Chen et al., 2009; Mohanta and Bae, 2015; MacLean et al., 2017; Yano et al., 2008). Downstream transcription factors, including Nodule Inception-like Proteins (NLPs), Nuclear Factor-Y (NF-Y), Lateral Organ Boundaries Domain 16 (LBD16), SHORT-ROOT (SHR)–SCARECROW (SCR) complex, and maize Histidine Kinase 1 (ZmHK1, CRE1 ortholog), regulate cortical and endodermal responses (Mohanta and Bae, 2015; Jin et al., 2016). Oxygen buffering by class-III/IV phytoglobins maintains low O_2_ niches for nitrogenase activity encoded by nifHDK (Guo et al., 2023a; de Bruijn, 2019). Furthermore, NH₃ is assimilated via the Glutamine Synthetase–Glutamate Synthase (GS/GOGAT) pathway and transported through ammonium transporters (AMTfs) into plant metabolism (Guo et al., 2023a; Wang et al., 2023; Mahmud et al., 2020). GS/GOGAT, glutamine synthetase–glutamate synthase pathway; AMTs, ammonium transporters.

The integration of GWAS, RNAi, CRISPR, cytological visualization, selective breeding, and microbiome engineering offers a comprehensive framework for enhancing diazotroph activity and BNF in Poaceae crops. Multi-omics integration with GWAS, in maize, predicts functional outputs like nitrogenase activity (Wallace et al., 2018). Microbiome engineering is advancing toward sophisticated consortia designs and incorporating helper strains to enhance diazotroph stability (Liu et al., 2024). Thus, reducing synthetic fertilizer use through enhanced BNF mitigates soil acidification, groundwater pollution, and greenhouse gas emissions, aligning with initiatives like the C_4_ Rice Project (Good and Beatty, 2011). However, the stability and ecological safety of SynCom remain a pressing challenge since field trials often face inoculant displacement by native soil microbiota, environmental variability, and regulatory barriers. A recent study highlighted the value of SynComs as an experimental model to better understand microbial interactions, which is necessary step towards ensuring long-term community resilience in the field (Fonseca-García et al., 2024). The integration of host genotype selection with multi-omics–guided SynCom design may enable the creation of tailored, durable microbial consortia that consistently enhance N fixation across agroecosystems. Challenges, such as complex plant–microbe–environment interactions, yield trade-offs, and regulatory frameworks necessitate multidisciplinary collaboration and field validation.

### 6.4 Optimization of cultivation practices

An alternative strategy for improving rhizosphere N fixation efficiency in Poaceae crops to develop highly efficient N-fixing rhizosphere environment during cultivation. This approach balances dynamic interactions among chemical inhibition, energy supply, and microbial functional expression. Controlling N fertilizer application, particularly by reducing ammonium inputs, can mitigate competitive inhibition of nitrogenase, essential for activating N fixation. The exogenous addition of straw, biochar, or organic fertilizers provides carbon sources (e.g., malate and sucrose) to support N-fixing bacterial metabolism and regulates the soil C/N ratio, thereby enhancing microbial activity. Additionally, simultaneous foliar application of Mo and Fe, key cofactors for nitrogenase, can mitigate metal availability limitations and boost nitrogenase activity.

Innovative modifications to traditional agronomic practices can develop low-oxygen micro-environments. For instance, intermittent irrigation (alternating between wet and dry conditions) reduces the levels of rhizosphere dissolved oxygen and maintains nitrogenase activity and functional threshold. No-tillage or reduced tillage practices preserve soil aggregate structure, thus stabilizing the ecological niche for N-fixing bacteria. Intercropping leguminous and Poaceae crops (e.g., maize-soybean) takes advantage of the “N spillover effect” of legume-associated rhizobia to boost N fixation in the rhizosphere of cereal crops. Furthermore, advanced bioaugmentation strategies, such as sodium alginate microspheres, whose slow-release properties enhance bacterial survival, encapsulate N-fixing bacteria in biodegradable materials. The implementation of the aforementioned measures can produce a synergistic effect. For example, combining N reduction with biochar application alleviates chemical inhibition and promotes the adhesion of N-fixing bacteria to the porous structure of biochar. The combination of no-tillage and intercropping optimizes the physical soil structure and biological interactions.

## 7 Challenges and future research directions

Cereal crops, the most important staple crops worldwide, are cultivated extensively across various regions. Although N fixation efficiency per unit is lower than legumes, ANF is widespread, suggesting considerable N-fixing potential. The roots, stems, and leaves of cereal crops have a diverse range of N-fixing bacteria. However, the interaction between host crops and associative N-fixing bacteria remains relatively weak, limiting the effectiveness of ANF in field applications. Enhancing and optimizing this associative relationship is crucial to fully realizing the N-fixing potential of cereal crops. Current research on BNF in these crops faces several challenges, including incomplete identification of N-fixing bacterial groups and functions and limited genetic understanding.

Furthermore, the interactions between N-fixing bacteria and other functional bacteria, fungi, and viruses are poorly understood, with unclear mechanisms of synergistic enhancement. The colonization processes of N-fixing bacteria in the rhizosphere, root interior, and aerial plant organs are scarcely characterized, and the mechanisms underlying microbe-plant interactions under different host genotypes and abiotic factors remain unclear. Therefore, future research should focus on verifying the genetic interaction networks between hosts and microbes for molecular improvement, validating the field stability and ecological safety of engineered bacterial strains, and addressing the high cost of large-scale implementation of agronomic measures. Plant-mediated microbiome manipulation in Poaceae crops offers holistic strategy to enhance diazotroph-driven BNF, leveraging genomic, molecular, cytological, breeding, and microbial engineering approaches. Rice, wheat, maize, and sugarcane serve as models and targets, enabling the design of cropping systems that are less fertilizer-dependent, more productive, and resilient, thus advancing sustainable food security in the era of climate change. Future studies should prioritize multi-omics-guided precision design, developing *in situ* N fixation monitoring technologies, and optimizing region-specific management strategies to bridge the gap between theoretical advancements and practical applications in the ANF for Poaceae crops.

## Footnotes

1
https://c4rice.com/
